# Complete genome sequence for the extremely thermophilic bacterium *Anaerocellum danielii* (DSM:8977)

**DOI:** 10.1128/mra.01229-23

**Published:** 2024-01-24

**Authors:** Mohamad J. H. Manesh, Ryan G. Bing, Daniel J. Willard, Michael W. W. Adams, Robert M. Kelly

**Affiliations:** 1 Department of Chemical and Biomolecular Engineering, North Carolina State University, Raleigh, North Carolina, USA; 2 Department of Biochemistry and Molecular Biology, University of Georgia, Athens, Georgia, USA; Portland State University, Portland, Oregon, USA

**Keywords:** *Anaerocellum danielii*, extreme thermophile, cellulose

## Abstract

The complete genome sequence of the extremely thermophilic bacterium *Anaerocellum* (*f. Caldicellulosiruptor*) *danielii* (DSM:8977) is reported here. *A. danielii* is a fermentative anaerobe and capable of lignocellulose degradation with potential applications in biomass degradation and production of chemicals and fuels from renewable feedstocks.

## ANNOUNCEMENT

*Anaerocellum* (*f. Caldicellulosiruptor*) *danielii* was isolated from a hot spring in Waimangu, New Zealand, and grows optimally at 75°C, with pH of 7.2 (1). Microorganisms from the order Caldicellulosiruptorales are capable of degrading lignocellulose and solubilizing carbohydrates and have potential for fuel and chemical production from renewable resources (2, 3). The previous *A. danielii* reference genome (ASM95572v1) (3) was not circularized. Here, the circularized genome sequence of *A. danielii* is reported, which consists of 2,822,094 bp.

*A. danielii* culture was procured previously from DSMZ and maintained as −80°C freezer stocks and grown for genome sequencing at 75°C in modified DSM516 medium, as previously described (4). Genomic DNA was extracted and purified from liquid cultures using the NEB Monarch Genomic DNA Purification Kit (New England Biolabs, USA) with the tissue lysis buffer and lysozyme. The gDNA was sequenced on a MinION Mk1B using an Oxford Nanopore Technologies (UK) R9.4.1 flow cell (FLO-MIN106D) without size selection. *A. danielli* gDNA was multiplexed and prepared for sequencing using the Oxford Nanopore Technologies Native Barcoding Kit (SQK-NBD112-24). During library preparation, DNA concentrations were quantified on a Qubit (v.4.0) fluorimeter using the 1× dsDNA HS Assay Kit (Invitrogen, Q33231). The sequencing run lasted 72 hours, with live GPU base-calling performed using MinKNOW (v.22.12.7) and Guppy (v.6.4.6) with the high-accuracy algorithm.

Initial trimming of barcodes from the reads was included in the Guppy base-calling workflow. Further filtering of the reads was performed with NanoFilt (v.2.8.0) (5) using a read quality cutoff of Q10 and a length cutoff of 1,000 bp. The filtered reads were assembled into a circular contig using Flye (v.2.9.1-b1780) (6). The final assembly was rotated to start at the *dnaA* gene using the “fixstart” command in Circlator (v.1.5.5) (7). For post-assembly processing, the filtered long reads were mapped against the rotated assembly and indexed using GraphMap (v.0.5.2) (8) and SAMtools (v.1.16.1) (9), respectively. The mapped reads were used for error correction through Pilon (v.1.24) (10) and polishing through Medaka (v.1.11.1) (Oxford Nanopore Technologies). QUAST (v.5.2.0) (11), Pilon (with --fix set to “none”) (10), and CheckM (v.1.2.2) (12) were used to generate assembly statistics, check for assembly errors, and assess the assembly quality. The final assembly was annotated using PGAP (v.2023-10-03.build7061) (13). All programs were run using default parameters expect where specified. A summary of assembly statistics is provided in Table 1.

**TABLE 1:** Statistics for *A. danielii* genome assemblies

	*Anaerocellum danielii*
Genome accession ID	CP139957
BioProject	PRJNA1046088
BioSample	SAMN38472127
Sequence Read Archive	SRR27048298
Number of reads	80,438
Total reads (bp)	478,059,903
Read n50 (bp)	2,822,094
Genome size (bp)	2,822,094
GC content (%)	35.74
Read coverage	158 x
Completeness (%)	99.26
Contamination (%)	0.00
Number of predicted genes	2,702

## Data Availability

Raw reads SRA, BioSample, BioProject, and genome accession numbers are included in Table 1 to access the data used for this project through National Center for Biotechnology Information depositions.
